# Tight Bounds for Joint Distribution Functions of Order Statistics Under *k*-Independence

**DOI:** 10.3390/e27121250

**Published:** 2025-12-11

**Authors:** Andrzej Okolewski, Barbara Blazejczyk-Okolewska

**Affiliations:** 1 Institute of Mathematics, Lodz University of Technology, 93-590 Lodz, Poland; 2 Division of Dynamics, Lodz University of Technology, 90-537 Lodz, Poland; okolbar@p.lodz.pl

**Keywords:** order statistics, dependence uncertainty, *k*-independent observations, distribution bounds, coherent systems with shared components

## Abstract

The present study investigates the problem of determining sharp bounds for key reliability and distributional characteristics associated with order statistics. We establish pointwise sharp two-sided bounds for linear combinations of joint distribution functions and joint reliability functions of selected order statistics based on *k*-independent and identically distributed random variables. The proposed framework is general and also applies to arbitrarily dependent observations. The obtained results provide exact bounds for the expected values of functions of order statistics corresponding to finite-valued random variables. Furthermore, the study yields the best possible upper and lower bounds for the joint reliability function of semicoherent systems with shared exchangeable *k*-independent components.

## 1. Introduction

Order statistics have found important applications in many diverse areas, including life testing and reliability, robust inference, statistical quality control, filtering theory, and signal and image processing (see, e.g., [1]). The properties of order statistics based on independent and identically distributed random variables and, to a large extent, on independent but non-identically distributed samples are well-established and have been extensively studied (see, e.g., [2,3]). Several classical results obtained for independent observations have been extended to dependent samples with prescribed joint distributions or partially known moments (see, e.g., [4,5,6,7,8,9,10,11]).

Research on the extremal properties of order statistic distributions under dependence uncertainty has primarily focused on cases where no restrictions are imposed on the interdependence of observations. In these frameworks, either the one-dimensional marginals are assumed to be known [12,13,14,15,16,17], or the distributions of the maxima (or minima) of all *k*-tuples are assumed to be identical and predefined (see [18,19]). Few studies have investigated the optimal estimation of distribution functions of order statistics from samples of dependent random variables with partially known dependence structures. Kemperman’s [20] analysis of *k*-independent, identically distributed observations yielded the first significant result in this field, providing a general approach for deriving pointwise sharp bounds for the distribution functions of order statistics and establishing the best-possible upper bounds for single-order statistics from pairwise independent observations. Extensions of Kemperman’s results to piecewise uniform marginal copulas and linear combinations of distribution functions of single-order statistics were presented in [21]. Mallows [22] made another significant contribution by considering three-element, two-independent samples with uniform marginals and constructing explicit extremal distributions that maximize the distribution function of the minimum. Furthermore, Okolewski [23] analyzed the extremal properties of order statistic distributions for dependent samples with partially specified multivariate marginals when the marginal copula diagonals up to a certain dimension are known.

The present study is concerned with determining pointwise sharp bounds for linear combinations of joint distribution functions and joint reliability functions of selected order statistics derived from identically distributed *k*-independent random variables. The problem is reformulated as a moment problem and solved using a geometric approach. The bounds established are significant because, in particular, they hold under minimal assumptions-specifically, for arbitrarily dependent random variables, without requiring knowledge of their joint dependence structure. In reliability theory, these results enable the derivation of best-possible lower and upper bounds for the reliability functions of semicoherent systems with shared, exchangeable components when the dependence structure among component lifetimes is completely unspecified or exhibits *k*-independence (see Remark 3). Additionally, the bounds provide precise expectation ranges for functions of order statistics when the underlying random variables take values in a finite set. In the case of possibly dependent random variables, these results may be applicable in the context of largest-claims (LC) reinsurance, where conservative bounds are essential for risk assessment and pricing under unknown dependence structures (see Remark 2).

This paper is organized as follows: Section 2 derives sharp distribution bounds for order statistics and illustrates their applications. Section 3 presents explicit examples that demonstrate the theoretical results.

## 2. Distribution Bounds for Order Statistics

Suppose we have *n* random variables $X_{1},\ldots ,X_{n}$, and order statistics $X_{1:n},\ldots ,X_{n:n}$ based on them. Let $x_{1}<\ldots <x_{q}$ be distinct real numbers. The number *q* may be greater than, equal to, or less than *n*. We consider multivariate marginal distribution functions

$$F_{j_{1},\ldots ,j_{m}}\left(x_{\ell_{1}},\ldots ,x_{\ell_{m}}\right)=P\left(X_{j_{1}:n}\leq x_{\ell_{1}},\ldots ,X_{j_{m}:n}\leq x_{\ell_{m}}\right)$$

of several order statistics $X_{j_{1}:n},\ldots ,X_{j_{m}:n}$ for some $1\leq j_{1}<\ldots <j_{m}\leq n$, evaluated at points belonging to the fixed finite set $\{x_{1},\ldots ,x_{q}\}.$ It suffices to consider only strictly increasing arguments $x_{\ell_{1}}<\ldots <x_{\ell_{m}}$, since otherwise redundant terms can be removed.

More generally, we study arbitrary linear combinations

(1) L(X):=∑m=1n∧q∑1≤j1<…<jm≤n1≤ℓ1<…<ℓm≤qcj1,…,jmℓ1,…,ℓmFj1,…,jm:n(xℓ1,…,xℓm)

involving multivariate marginal distribution functions of order statistics evaluated at the elements of the finite set $\left\{x_{1},\ldots ,x_{q}\right\}$. Here, n∧q=min(n,q). By setting some coefficients $c_{j_{1},\ldots ,j_{m}}^{\ell_{1},\ldots ,\ell_{m}}=0$, various reductions are possible. The value L(X) is uniquely determined when the distribution of the vector $X=(X_{1},\ldots ,X_{n})$ is fully known. When only partial information on X is available, L(X) takes values within a certain range.

In statistical theory, it is well known that for a set of three or more random variables to be mutually independent, pairwise independence is necessary but not sufficient. A classic example of three discrete variables that are pairwise independent but not mutually independent, frequently cited in the statistical literature, is due to S. Bernstein (see Cramér [24]). For an overview of generalizations of this example to cases with more than three variables and continuous distributions, see [25]. In this work, the author constructs an infinite sequence of random variables such that the components of any proper subset are independent if and only if the size of the subset is less than or equal to a fixed positive integer *k*. Such random variables are called *k*-independent. *k*-independence is a powerful tool because it provides a balance between full independence and arbitrary dependence. It is often used to reduce the amount of randomness required in probabilistic algorithms, for example, when weaker sources of randomness are sufficient for analyses that use Chernoff–Hoeffding bounds under limited independence, such as the analysis of randomized algorithms for random sampling (see [26]).

We are interested in determining sharp lower and upper bounds for L(X) over the set of all possible distributions of *k*-independent vectors X having the same one-dimensional marginal distribution *F*. Here, X is *k*-independent if every *k*-tuple $\left(X_{j_{1}},\ldots ,X_{j_{k}}\right)$ is independent (for k>1), while in the case k=1, the components may be arbitrarily dependent. Our approach transforms this problem into a moment problem based on Kemperman’s characterization of *k*-independence [20] [Theorem 1]: A random vector $Y=(Y_{0},Y_{1},\ldots ,Y_{q})$ has the same distribution as the vector $B=(B_{0},B_{1},\ldots ,B_{q})$, where

(2) $$\begin{array}{c} B_{p}=\sum_{j=1}^{n}1\left(x_{p}<X_{j}\leq x_{p+1}\right),\;p=0,1,\ldots ,q, \end{array}$$

associated to some *k*-independent X, if and only if Y takes values in the set

$$\sum_{q}\left[n\right]=\left\{\left(y_{0},y_{1},\ldots ,y_{q}\right)\in Z_{+}^{q+1}:\sum_{p=0}^{q}y_{p}=n\right\}$$

and satisfies the moment conditions

(3) $$\begin{array}{c} E\left[\prod_{p=0}^{q}{\left(Y_{p}\right)}_{i_{p}}\right]={\left(n\right)}_{r_{i}}\prod_{p=0}^{q}\pi_{p}^{i_{p}}\;\mathrm{for}\;\mathrm{all}\;\left(i_{0},\ldots ,i_{q}\right)\in I_{k}^{q+1}, \end{array}$$

where 1(s)=1 if *s* is true and 1(s)=0 otherwise, $Z_{+}$ is the set of non-negative integers, ${\left(s\right)}_{j}=s\left(s-1\right)\ldots \left(s-j+1\right)$ denotes the falling factorial, indices satisfy $r_{i}=\sum_{p=0}^{q}i_{p}$, and

(4) $$\begin{array}{c} I_{k}^{q+1}=\left\{\left(i_{0},\ldots ,i_{q}\right)\in Z_{+}^{q+1}:0<\sum_{p=0}^{q}i_{p}\leq k\right\}, \end{array}$$

with probabilities $\pi_{p}=F\left(x_{p+1}\right)-F\left(x_{p}\right)$, assuming $x_{0}=-\infty$ and $x_{q+1}=+\infty$.

Throughout, we assume that

(5) $$\begin{array}{c} F\left(x_{p+1}\right)-F\left(x_{p}\right)>0,\;p=0,1,\ldots ,q, \end{array}$$

to avoid trivial cases.

Our main result states that the bounds on L(X) can be expressed as values of certain known functions, which depend solely on the coefficients c, evaluated at points determined exclusively by the values x. In essence, these bounds take the same form as those for linear combinations of distribution functions of individual order statistics from possibly dependent observations, as established in [16].

Before formulating the result, we introduce some notation and terminology. Since $\sum_{p=0}^{q}Y_{p}=\sum_{p=0}^{q}B_{p}=n$, it suffices to require condition (3) only for $i_{q}=0$ (cf. Kemperman [20] [Remark]). Denote by $\delta^{1},\ldots ,\delta^{\vartheta }$ the ϑ=∑j=1k(q+j−1j) elements of the set $I_{k}^{q}$, which consists of all orders not exceeding *k* of factorial moments of the random vector

$$Y^{-}=\left(Y_{0},\ldots ,Y_{q-1}\right)$$

taking values in the set

(6) $$\begin{array}{c} U_{n}^{q}=\left\{\left(u_{0},u_{1},\ldots ,u_{q-1}\right)\in Z_{+}^{q}:0\leq \sum_{p=0}^{q-1}u_{p}\leq n\right\}. \end{array}$$

Define the vector function

$$\theta_{k}\left(u\right)=\left(\theta_{k}^{1}\left(u\right),\ldots ,\theta_{k}^{\vartheta }\left(u\right)\right),$$

where

$$\theta_{k}^{j}\left(u\right)=\prod_{p=0}^{q-1}{\left(u_{p}\right)}_{\delta_{p}^{j}}$$

for $u=\left(u_{0},u_{1},\ldots ,u_{q-1}\right)\in U_{n}^{q}$. Each component $\theta_{k}^{j}\left(u\right)$ represents a possible value of the product $\prod_{p=0}^{q-1}{\left(Y_{p}\right)}_{\delta_{p}^{j}}$ for a vector $\delta^{j}$ from $I_{k}^{q}$. Accordingly, the coordinates of $\theta_{k}\left(u\right)$ enumerate the possible values of such products for all vectors in $I_{k}^{q}$.

Let $M_{k}$ be the compact and convex set of all possible moments $E\theta_{k}\left(Y^{-}\right)$, where the distribution of $Y^{-}$ varies over all probability distributions on $U_{n}^{q}$. Clearly, we have

$$M_{k}=\mathrm{conv}\Gamma_{k},$$

where

$$\Gamma_{k}=\left\{\theta_{k}\left(u\right):u\in U_{n}^{q}\right\},$$

and convA denotes the convex hull of the set *A*.

We now define two functions necessary to state our results. For any function $\varphi :U_{n}^{q}\to R$, define

$$C_{\varphi }:M_{k}\to R\;\mathrm{and}\;C^{\varphi }:M_{k}\to R,$$

to be, respectively, the greatest convex function such that

$$C_{\varphi }\left(\theta_{k}\left(u\right)\right)\leq \varphi \left(u\right)\;\mathrm{for}\;\mathrm{all}\;u\in U_{n}^{q},$$

and the smallest concave function such that

$$C^{\varphi }\left(\theta_{k}\left(u\right)\right)\geq \varphi \left(u\right)\;\mathrm{for}\;\mathrm{all}\;u\in U_{n}^{q}.$$

**Theorem 1.**  *Let 1≤k≤n, n≥2, and q≥1 be fixed integers. Let $x_{1}<\ldots <x_{q}$ and $c_{j_{1},\ldots ,j_{m}}^{\ell_{1},\ldots ,\ell_{m}}$ be fixed real numbers, where m=1,…,n∧q, $1\leq j_{1}<\ldots <j_{m}\leq n$, and $1\leq \ell_{1}<\ldots <\ell_{m}\leq q$.*

*(i) If $X=(X_{1},\ldots ,X_{n})$ is a vector of k-independent random variables with a common distribution function F satisfying condition (5), then the following bounds hold:*

(7) $$\begin{array}{c} C_{\varphi }\left(m\left(F\left(x_{1}\right),\ldots ,F\left(x_{q}\right)\right)\right)\leq L\left(X\right)\leq C^{\varphi }\left(m\left(F\left(x_{1}\right),\ldots ,F\left(x_{q}\right)\right)\right), \end{array}$$

*where*

(8) φ(u0,…,uq−1)=∑m=1n∧q∑1≤j1<…<jm≤n1≤ℓ1<…<ℓm≤qcj1,…,jmℓ1,…,ℓm1∑p=0ℓ1−1up≥j1,…,∑p=0ℓm−1up≥jm

*for $\left(u_{0},\ldots ,u_{q-1}\right)\in U_{n}^{q}$ and*

(9) $$\begin{array}{c} m\left(t\right)=(m_{1}\left(t\right),\ldots ,m_{\vartheta }\left(t\right)) \end{array}$$

*with*

$$m_{j}\left(t\right)=m_{j}\left(t_{1},\ldots ,t_{q}\right)={\left(n\right)}_{r_{j}}\prod_{p=0}^{q-1}{\left(t_{p+1}-t_{p}\right)}^{\delta_{p}^{j}},$$

*in which $0=t_{0}<t_{1}<\ldots <t_{q}<1$ and $r_{j}=\sum_{p=0}^{q-1}\delta_{p}^{j}.$*

*(ii) Moreover, for any distribution function F that satisfies condition (5), there exist k-independent random variables $X_{1},\ldots ,X_{n}$ with common distribution function F such that equality is attained in the first (resp. second) inequality (7).*

**Proof.**  From Kemperman’s [20] [Theorem 1, Remark] characterization of *k*-independence, it follows that a random vector $Y^{-}=\left(Y_{0},Y_{1},\ldots ,Y_{q-1}\right)$ has the same distribution as the random vector $B^{-}=\left(B_{0},B_{1},\ldots ,B_{q-1}\right)$, associated as in (2) to some *k*-independent random vector $X=(X_{1},\ldots ,X_{n})$ with marginals *F*, if and only if $Y^{-}$ takes values in $U_{n}^{q}$ and satisfies the condition

(10) $$\begin{array}{c} E\left[\prod_{p=0}^{q-1}{\left(Y_{p}\right)}_{i_{p}}\right]={\left(n\right)}_{r_{i}}\prod_{p=0}^{q-1}\pi_{p}^{i_{p}}\;\mathrm{for}\;\mathrm{all}\;\left(i_{0},i_{1},\ldots ,i_{q-1}\right)\in I_{k}^{q} \end{array}$$

with $r_{i}=\sum_{p=0}^{q-1}i_{p}$, $I_{k}^{q}$ as in (4), and $\pi_{p}=F\left(x_{p+1}\right)-F\left(x_{p}\right)$. Observe that condition (10) can be rewritten in the form

(11) $$\begin{array}{c} E\theta_{k}\left(Y^{-}\right)=m\left(t\right) \end{array}$$

with m(t) as in (9) and $t=(F\left(x_{1}\right),\ldots ,F\left(x_{q}\right))$.

For any given function ψ, minimizing (resp. maximizing) the moment $E\psi (B^{-})$ is equivalent to minimizing (resp. maximizing) $E\psi (Y^{-})$, subject to the constraints that $Y^{-}$ takes values in $U_{n}^{q}$ and satisfies the moment condition (11). Since $X_{s:n}\leq x$ if and only if $\sum_{j=1}^{n}1\left(X_{j}\leq x\right)\geq s$ and $\sum_{j=1}^{n}1\left(X_{j}\leq x_{\ell }\right)=\sum_{p=0}^{\ell -1}B_{p}$, by (1) and (2), one has

$$L\left(X\right)=\sum_{m=1}^{n\wedge q}\sum_{\begin{array}{c} 1\leq j_{1}<\ldots <j_{m}\leq n\\ 1\leq \ell_{1}<\ldots <\ell_{m}\leq q \end{array}}c_{j_{1},\ldots ,j_{m}}^{\ell_{1},\ldots ,\ell_{m}}\;P\left(\overset{m}{\underset{\nu =1}{⋂}}\left\{\sum_{p=0}^{\ell_{\nu }-1}B_{p}\geq j_{\nu }\right\}\right).$$

Hence, determining bounds on L(X) reduces to minimizing (or maximizing)

$$T\left(Y^{-}\right):=\sum_{m=1}^{n\wedge q}\sum_{\begin{array}{c} 1\leq j_{1}<\ldots <j_{m}\leq n\\ 1\leq \ell_{1}<\ldots <\ell_{m}\leq q \end{array}}c_{j_{1},\ldots ,j_{m}}^{\ell_{1},\ldots ,\ell_{m}}\;P\left(\overset{m}{\underset{\nu =1}{⋂}}\left\{\sum_{p=0}^{\ell_{\nu }-1}Y_{p}\geq j_{\nu }\right\}\right),$$

subject to $Y^{-}\in U_{n}^{q}$ and the moment constraint (11).

We solve the latter problem using a geometric approach. Note that $L\left(X\right)=E\varphi \left(B^{-}\right)$, where φ is given by (8). Recall that $M_{k}$ is the compact set of all possible moment points $E\theta_{k}\left(Y^{-}\right)\in R^{\vartheta }$ where the distribution of $Y^{-}$ varies over all distributions on $U_{n}^{q}$.

Consider the auxiliary function

$${\tilde{\theta }}_{k}\left(u\right)=\left(\theta_{k}^{1}\left(u\right),\ldots ,\theta_{k}^{\vartheta }\left(u\right),\varphi \left(u\right)\right).$$

Since

$${\tilde{M}}_{k}=\mathrm{conv}\left\{{\tilde{\theta }}_{k}\left(u\right):u\in U_{n}^{q}\right\}$$

is the compact set of all possible moment points $E{\tilde{\theta }}_{k}\left(Y^{-}\right)\in R^{\vartheta +1}$, where the random vector $Y^{-}$ takes its values in $U_{n}^{q},$ the line segment

$${\tilde{M}}_{k}\cap \left\{\left(m\left(t\right),\rho \right):\rho \in R\right\}$$

represents the range of possible moment points $E{\tilde{\theta }}_{k}\left(Y^{-}\right)\in R^{\vartheta +1},$ where the distribution of $Y^{-}$ varies over all distributions on $U_{n}^{q}$ satisfying the condition $E\theta_{k}\left(Y^{-}\right)=m\left(t\right).$ The lower (resp. upper) endpoint belongs to the lower (resp. upper) envelope of ${\tilde{M}}_{k}$, defined by $\left(y,C_{\varphi }\left(y\right)\right)$ (resp. $\left(y,C^{\varphi }\left(y\right)\right)$ for $y\in M_{k}$, providing the sharp lower (resp. upper) bound on L(X) (cf. [27]).

This completes the proof of (i). Statement (ii) follows directly from [20] [Theorem 1]. □

**Remark 1.** *(i) The bounds for multivariate marginal distribution functions of order statistics provided by Theorem 1 are new even in the case k=1, that is, when the $X_{j}$’s are arbitrarily dependent identically distributed random variables.*

*(ii) Fix j∈{1,…,n} and let q=1, k=2, and $c_{j_{1},\ldots ,j_{m}}^{\ell_{1},\ldots ,\ell_{m}}=1\left(m=1,j_{1}=j,\ell_{1}=1\right)$ in (7). Then we recover Kemperman’s [20] bounds for single-order statistics from pairwise independent observations. If we fix $c_{1},\ldots ,c_{n}\in R$ and k∈{1,…,n−1}, and take q=1 and $c_{j_{1},\ldots ,j_{m}}^{\ell_{1},\ldots ,\ell_{m}}=c_{j_{1}}1\left(m=1,\ell_{1}=1\right)$ in (7), we obtain more explicit expressions for the bounds [21] Equation (28) for linear combinations of distribution functions of single-order statistics from k-independent observations.*

*(iii) From the proof of Theorem 1 it follows that the range of possible values of L(X) over all possible distributions of k-independent vectors X with one-dimensional marginals F is equal to the interval $[C_{\varphi }\left(m\left(t\right)\right),C^{\varphi }\left(m\left(t\right)\right)],$ where $t=(F\left(x_{1}\right),\ldots ,F\left(x_{q}\right).$*

**Remark 2.**  *The pointwise sharp distribution bounds (7) do not generally yield sharp expectation bounds (cf. [20]). However, the bounds obtained in this way may be accurate in certain particular cases. Indeed, suppose that q∈{2,3,…} and the k-independent $X_{j}$’s have a common piecewise constant distribution function F with jumps at points $-\infty <x_{1}<\ldots <x_{q}<\infty .$ For example, for any fixed $1\leq j_{1}<j_{2}\leq n$ and a function $h:R^{2}\to R$, we have*

$$\begin{array}{c} Eh\left(X_{j_{1}:n},X_{j_{2}:n}\right)=\sum_{\ell_{1}=1}^{q}\sum_{\ell_{2}=1}^{q}h\left(x_{\ell_{1}},x_{\ell_{2}}\right)P\left(X_{j_{1}:n}=x_{\ell_{1}},X_{j_{2}:n}=x_{\ell_{2}}\right), \end{array}$$

*which can be expressed as*

$$\begin{array}{c} Eh\left(X_{j_{1}:n},X_{j_{2}:n}\right)=\sum_{\ell_{2}=1}^{q}\left(\sum_{\ell_{1}=\ell_{2}}^{q}\;h_{\ell_{1}\ell_{2}}\right)F_{j_{2}:n}\left(x_{\ell_{2}}\right)+\sum_{\ell_{1}=1}^{q-1}\sum_{\ell_{2}=\ell_{1}+1}^{q}\;h_{\ell_{1}\ell_{2}}F_{j_{1},j_{2}:n}\left(x_{\ell_{1}},x_{\ell_{2}}\right), \end{array}$$

*where*

$$\begin{array}{ccc} h_{\ell_{1}\ell_{2}} & = & h\left(x_{\ell_{1}},x_{\ell_{2}}\right)-h\left(x_{\ell_{1}},x_{\ell_{2}+1}\right)1\left(\ell_{2}<q\right)-h\left(x_{\ell_{1}+1},x_{\ell_{2}}\right)1\left(\ell_{1}<q\right)\\ & & +\;h\left(x_{\ell_{1}+1},x_{\ell_{2}+1}\right)1\left(\ell_{1}<q,\ell_{2}<q\right). \end{array}$$

*Thus, applying (7) yields two-sided attainable bounds for $Eh(X_{j_{1}:n},X_{j_{2}:n})$, which are expressed in terms of the points $x_{1},\ldots ,x_{q}$ and the values of the distribution function $F\left(x_{1}\right),\ldots ,F\left(x_{q}\right).$ Sharp expectation bounds for L-statistics from arbitrarily dependent observations (i.e., for k=1) were presented in [16]. Note that for $h\left(s_{1},s_{2}\right)=s_{1}+s_{2}$, the expectation of $h(X_{n-1:n},X_{n:n})$ represents the net premium for the largest-claims (LC) reinsurance of the two largest claims in an individual model of homogeneous, and in particular arbitrarily dependent, risks (cf. [28]). In the context of LC reinsurance, conservative bounds are essential for risk assessment and pricing under unknown dependence structures.*

Our next objective is to establish an analogue of Theorem 1 for linear combinations of joint reliability functions associated with selected order statistics, defined by

(12) L¯(X):=∑m=1n∧q∑1≤j1<…<jm≤n1≤ℓ1<…<ℓm≤qcj1,…,jmℓ1,…,ℓmF¯j1,…,jm:n(xℓ1,…,xℓm),

where $x_{1}<\ldots <x_{q},$ $c_{j_{1},\ldots ,j_{m}}^{\ell_{1},\ldots ,\ell_{m}}$ are fixed real numbers, and ${\bar{F}}_{j_{1},\ldots ,j_{m}:n}\left(x_{\ell_{1}},\ldots ,x_{\ell_{m}}\right)$$:=P(X_{j_{1}:n}>x_{\ell_{1}},\ldots ,X_{j_{m}:n}>x_{\ell_{m}}).$

**Theorem 2.**  *Under the assumptions and notation of Theorem 1, the following statements hold.*

*(i) Let $X=(X_{1},\ldots ,X_{n})$ be a vector of k-independent random variables with a common distribution function F fulfilling condition (5). Then*

(13) $$\begin{array}{c} C_{\bar{\varphi }}\left(m\left(F\left(x_{1}\right),\ldots ,F\left(x_{q}\right)\right)\right)\leq \bar{L}\left(X\right)\leq C^{\bar{\varphi }}\left(m\left(F\left(x_{1}\right),\ldots ,F\left(x_{q}\right)\right)\right), \end{array}$$

*where*

φ¯(u0,…,uq−1)=∑m=1n∧q∑1≤j1<…<jm≤n1≤ℓ1<…<ℓm≤qcj1,…,jmℓ1,…,ℓm1∑p=0ℓ1−1up<j1,…,∑p=0ℓm−1up<jm.

*(ii) Moreover, for any distribution function F satisfying condition (5), there exist k-independent random variables $X_{1},\ldots ,X_{n}$ with distribution function F for which the lower (resp. upper) bound in (13) is attained.*

**Proof.**  Combining (12) with the fact that $X_{s:n}>x$ if and only if $\sum_{j=1}^{n}1\left(X_{j}\leq x\right)<s$, we obtain

L¯(X)=∑m=1n∧q∑1≤j1<…<jm≤n1≤ℓ1<…<ℓm≤qcj1,…,jmℓ1,…,ℓmP⋂ν=1m∑p=0ℓν−1Bp<jν.

An analysis analogous to that in the proof of Theorem 1 shows that determining bounds on $\bar{L}\left(X\right)$ is equivalent to minimizing (maximizing) the quantity

T¯(Y−):=∑m=1n∧q∑1≤j1<…<jm≤n1≤ℓ1<…<ℓm≤qcj1,…,jmℓ1,…,ℓmP⋂ν=1m∑p=0ℓν−1Yp<jν,

where the vector $Y^{-}=\left(Y_{0},Y_{1},\ldots ,Y_{q-1}\right)$ takes values in $U_{n}^{q}$ and satisfies condition (11). The proof is completed by adapting the second part of the proof of Theorem 1 with L(X) replaced by $\bar{L}\left(X\right)$ and φ replaced by $\bar{\varphi }.$ □

**Remark 3.**  *If the $X_{j}$’s are not only k-independent but also non-negative, exchangeable and have no ties, then (13) provides sharp bounds for the joint reliability function ${\bar{F}}_{s}$ of any pair of semi-coherent systems based on common components. Specifically,*

$${\bar{F}}_{s}\left(z_{1},z_{2}\right)=\sum_{j_{1}=1}^{n}\sum_{j_{2}=1}^{n}s_{j_{1}j_{2}}{\bar{F}}_{j_{1},j_{2}:n}\left(z_{1},z_{2}\right),\;z_{1},z_{2}\geq 0,$$

*where $s={\left(s_{j_{1}j_{2}}\right)}_{1\leq j_{1},j_{2}\leq n}$ is a probability matrix of order n depending solely on the system structure, known as the structure signature of the system (cf. [29]). To see this, observe that*

F¯s(z1,z2)=∑1≤j1≤n1≤ℓ1≤2cj1ℓ1F¯j1:n(xℓ1)+∑1≤j1<j2≤n1≤ℓ1<ℓ2≤2cj1,j2ℓ1,ℓ2F¯j1,j2:n(xℓ1,xℓ2),

*where $x_{1}=\min\left(z_{1},z_{2}\right)$, $x_{2}=\max\left(z_{1},z_{2}\right),$*

$$\begin{array}{c} c_{j_{1}}^{\ell_{1}}=\left[s_{j_{1}j_{1}}+\sum_{j=j_{1}+1}^{n}\left(s_{jj_{1}}1\left(z_{1}\leq z_{2}\right)+s_{j_{1}j}1\left(z_{1}\geq z_{2}\right)\right)\right]1\left(\ell_{1}=2\right) \end{array}$$

*and*

$$c_{j_{1}j_{2}}^{\ell_{1},\ell_{2}}=s_{j_{1}j_{2}}1\left(z_{1}<z_{2}\right)+s_{j_{2}j_{1}}1\left(z_{1}>z_{2}\right).$$

*Sharp bounds on the lifetime distributions and expectations of a single system with arbitrarily dependent exchangeable component lifetimes $X_{1},\ldots ,X_{n}$ were derived in [30].*

**Remark 4.**  *Theorems 1 and 2 reduce the problem of determining distribution bounds for order statistics from dependent observations to finding the convex hull of a finite set of points in $R^{\nu +1}$, where ν=∑j=1k(q+j−1j). Computing the convex hull is a fundamental step in many practical problems, including statistical tasks such as robust estimation, isotonic regression, and clustering (see [31]). Even in moderate dimensions, such as 10 or 20, convex hull computation can be challenging. Knowledge of specific properties of the convex hull in a given problem, for example, the presence of symmetry, can be helpful (see [32]). A comprehensive overview of algorithms and methods for determining the convex hull of a finite set of points in $R^{d}$ is provided in [33], along with a detailed discussion of their respective advantages, disadvantages, and recommended applications.*

## 3. Explicit Bounds for Possibly Dependent Observations

We will derive explicit sharp lower and upper bounds for the joint distribution function and joint reliability function of any pair of order statistics based on arbitrarily dependent observations.

**Proposition 1.**  *Let F be a given distribution function, and let $1\leq j_{1}<j_{2}\leq n$ and $x_{1}<x_{2}$ be such that $0<F\left(x_{1}\right)<F\left(x_{2}\right)<1.$ If $X=(X_{1},\ldots ,X_{n})$ is a vector of possibly dependent random variables with common distribution function F, then*

$$\begin{array}{c} F_{j_{1},j_{2}:n}\left(x_{1},x_{2}\right)\leq \min\left\{\frac{n}{j_{1}}F\left(x_{1}\right),\frac{n}{j_{2}}F\left(x_{2}\right),1\right\} \end{array}$$

*and*

$$\begin{array}{c} F_{j_{1},j_{2}:n}\left(x_{1},x_{2}\right)\geq \max\left\{H_{j_{1},j_{2}:n}\left(F\left(x_{1}\right),F\left(x_{2}\right)\right),0\right\} \end{array}$$

*with*

(14) $$\begin{array}{c} H_{j_{1},j_{2}:n}\left(z_{1},z_{2}\right)=\frac{\left(n-j_{2}+1\right)nz_{1}+\left(j_{2}-j_{1}\right)nz_{2}-\left(j_{2}-1\right)\left(n-j_{1}+1\right)}{\left(n-j_{1}+1\right)\left(n-j_{2}+1\right)}, \end{array}$$

*and both the bounds are sharp.*

**Proof.**  We apply Theorem 1 with parameters k=1, q=2, and n≥2. Let F, $1\leq j_{1}<j_{2}\leq n,$ and $x_{1}<x_{2}$ be such that $0<F\left(x_{1}\right)<F\left(x_{2}\right)<1.$ Then, we have that ϑ=2, $I_{1}^{2}=\left\{\delta^{1},\delta^{2}\right\},$ where $\delta^{1}=\left(1,0\right)$ and $\delta^{2}=\left(0,1\right),$ while

$$U_{n}^{2}=\left\{u=\left(u_{0},u_{1}\right):u_{0},u_{1}\in Z_{+};u_{0}+u_{1}\leq n\right\}.$$

Moreover, $m\left(t\right)=(m_{1}\left(t\right),m_{2}\left(t\right)),$ in which $t=\left(t_{1},t_{2}\right):=\left(F\left(x_{1}\right),F\left(x_{2}\right)\right),$ with $m_{1}\left(t\right)=nt_{1}>0$ and $m_{2}\left(t\right)=n\left(t_{2}-t_{1}\right)>0,$ and

$$M_{2}=\mathrm{conv}\left\{\theta_{2}\left(u\right):u=\left(u_{0},u_{1}\right)\in U_{n}^{2}\right\}$$

with $\theta_{2}\left(u\right)=u.$ Clearly, $m\left(t\right)\in M_{2}.$

**Figure 1:**
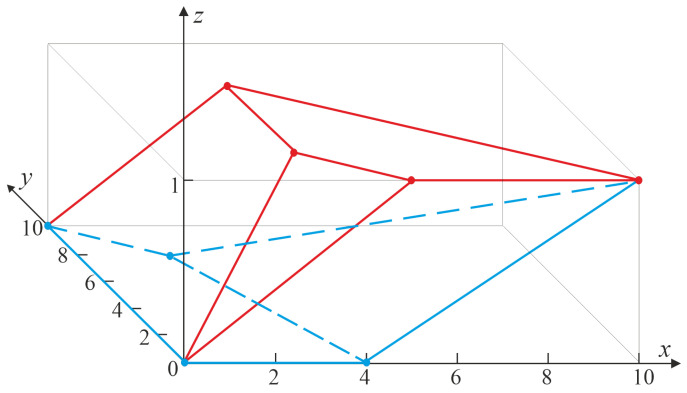
The set ${\tilde{M}}_{2}$ corresponding to $F_{3,5:10}$.

To determine the bounds $C_{\varphi }\left(m\left(t\right)\right)$ and $C^{\varphi }\left(m\left(t\right)\right)$ on $F_{j_{1},j_{2}:n}\left(x_{1},x_{2}\right),$ it suffices to find the lower envelope $\left\{\left(y,C_{\varphi }\left(y\right)\right):y\in M_{2}\right\}$ and the upper envelope $\left\{\left(y,C^{\varphi }\left(y\right)\right):y\in M_{2}\right\}$ of the set ${\tilde{M}}_{2}=\mathrm{conv}{\tilde{\Gamma }}_{2},$ where ${\tilde{\Gamma }}_{2}=\left\{{\tilde{\theta }}_{2}\left(u\right):u\in U_{n}^{2}\right\},$ ${\tilde{\theta }}_{2}\left(u_{0},u_{1}\right)=\left(u_{0},u_{1},\varphi \left(u_{0},u_{1}\right)\right),$ and $\varphi \left(u_{0},u_{1}\right)=1\left(u_{0}\geq j_{1},\;u_{0}+u_{1}\geq j_{2}\right)$. Figure 1 illustrates the set ${\tilde{M}}_{2}$ for the specific case n=10, $j_{1}=3$, and $j_{2}=5$. Notably, the convex hulls of the elements in the underlying set $\tilde{\Gamma_{2}}$ with the third coordinate equal to 0 and 1, respectively, form the lower (depicted in blue) and upper (depicted in red) faces of the polyhedron ${\tilde{M}}_{2}$, which are parallel to the *x*- and *y*-axes. The vertices of these faces constitute the set of extreme points of $\tilde{\Gamma_{2}}$, meaning that none of these points can be represented as a convex combination of other feasible points in the set.

Observe that, in the general case, the set $\mathrm{ext}{\tilde{M}}_{2}$ of extreme points of $\tilde{\Gamma_{2}}$, and thus also of ${\tilde{M}}_{2}$, is given by

$$\{{\tilde{\theta }}_{2}\left(u^{\left(0\right)}\right),{\tilde{\theta }}_{2}\left(u^{\left(1\right)}\right),\ldots ,{\tilde{\theta }}_{2}\left(u^{\left(7\right)}\right)\},$$

where $u^{\left(0\right)}=\left(0,0\right),$ $u^{\left(1\right)}=\left(n,0\right),$ $u^{\left(2\right)}=\left(0,n\right),$ $u^{\left(3\right)}=\left(j_{1},j_{2}-j_{1}\right),$ $u^{\left(4\right)}=\left(j_{2},0\right)$, $u^{\left(5\right)}=\left(j_{1},n-j_{1}\right),$ $u^{\left(6\right)}=\left(j_{1}-1,n-j_{1}+1\right)$ and $u^{\left(7\right)}=\left(j_{2}-1,0\right).$ Each of these points corresponds to a vertex of the convex hull of feasible integer triplets. In particular, none of them can be expressed as a convex combination of other feasible points in ${\tilde{M}}_{2}$. Therefore, they precisely constitute the extreme points that determine the boundary of the convex set.

Consequently,

$$\begin{array}{ccc} \mathrm{ext}{\tilde{M}}_{2} & = & \{\left(0,0,0\right),\left(n,0,1\right),\left(0,n,0\right),\left(j_{1},j_{2}-j_{1},1\right),\left(j_{2},0,1\right),\\ & & \;\;\left(j_{1},n-j_{1},1\right),\left(j_{1}-1,n-j_{1}+1,0\right),\left(j_{2}-1,0,0\right)\}. \end{array}$$

We first derive the upper bounds. Observe that

$$\begin{array}{c} M_{2}=M_{2}^{\left(1\right)}\cup M_{2}^{\left(2\right)}\cup M_{2}^{\left(3\right)} \end{array}$$

and the upper envelope $\mathrm{uenv}{\tilde{M}}_{2}$ of ${\tilde{M}}_{2}$ is given by

$$\begin{array}{c} \mathrm{uenv}{\tilde{M}}_{2}={\tilde{U}}_{2}^{\left(1\right)}\cup {\tilde{U}}_{2}^{\left(2\right)}\cup {\tilde{U}}_{2}^{\left(3\right)}, \end{array}$$

where

$$\begin{array}{ccc} M_{2}^{\left(1\right)} & = & \mathrm{conv}\{u^{\left(0\right)},u^{\left(3\right)},u^{\left(4\right)}\},\\ M_{2}^{\left(2\right)} & = & \mathrm{conv}\{u^{\left(0\right)},u^{\left(2\right)},u^{\left(3\right)},u^{\left(5\right)}\},\\ M_{2}^{\left(3\right)} & = & \mathrm{conv}\{u^{\left(1\right)},u^{\left(3\right)},u^{\left(4\right)},u^{\left(5\right)}\} \end{array}$$

and

$$\begin{array}{ccc} {\tilde{U}}_{2}^{\left(1\right)} & = & \mathrm{conv}\{{\tilde{\theta }}_{2}\left(u^{\left(0\right)}\right),{\tilde{\theta }}_{2}\left(u^{\left(3\right)}\right),{\tilde{\theta }}_{2}\left(u^{\left(4\right)}\right)\},\\ {\tilde{U}}_{2}^{\left(2\right)} & = & \mathrm{conv}\{{\tilde{\theta }}_{2}\left(u^{\left(0\right)}\right),{\tilde{\theta }}_{2}\left(u^{\left(2\right)}\right),{\tilde{\theta }}_{2}\left(u^{\left(3\right)}\right),{\tilde{\theta }}_{2}\left(u^{\left(5\right)}\right)\},\\ {\tilde{U}}_{2}^{\left(3\right)} & = & \mathrm{conv}\{{\tilde{\theta }}_{2}\left(u^{\left(1\right)}\right),{\tilde{\theta }}_{2}\left(u^{\left(3\right)}\right),{\tilde{\theta }}_{2}\left(u^{\left(4\right)}\right),{\tilde{\theta }}_{2}\left(u^{\left(5\right)}\right)\}. \end{array}$$

Figure 1 shows the outline of the upper envelope of the set ${\tilde{M}}_{2}$ for n=10, $j_{1}=3,$ and $j_{2}=5$, indicated in red.

We distinguish three cases: If m(t)$\in M_{2}^{\left(1\right)},$ then the restriction ${\tilde{U}}_{2}^{\left(1\right)}$ of $\mathrm{uenv}{\tilde{M}}_{2}$ to $M_{2}^{\left(1\right)}$ lies on the plane in $R^{3}$ described by the equation

$$z_{1}+z_{2}-j_{2}z_{3}=0.$$

By (7), the third coordinate of the intersection point of the line {(m(t),ρ):ρ∈R} with ${\tilde{U}}_{2}^{\left(1\right)}$ provides the desired upper bound:

$$\begin{array}{c} F_{j_{1},j_{2}:n}\left(x_{1},x_{2}\right)\leq \frac{n}{j_{2}}F\left(x_{2}\right) \end{array}$$

since $C^{\varphi }\left(z_{1},z_{2}\right)=\left(z_{1}+z_{2}\right)/j_{2}$.

If $m\left(t\right)\in M_{2}^{\left(2\right)},$ then the restriction of $\mathrm{uenv}{\tilde{M}}_{2}$ to $M_{2}^{\left(2\right)}$ equals ${\tilde{U}}_{2}^{\left(2\right)}.$ Any triple $\left(z_{1},z_{2},z_{3}\right)\in {\tilde{U}}_{2}^{\left(2\right)}$ satisfies the equation

$$z_{1}-j_{1}z_{3}=0,$$

yielding the upper bound

$$\begin{array}{c} F_{j_{1},j_{2}:n}\left(x_{1},x_{2}\right)\leq \frac{n}{j_{1}}F\left(x_{1}\right). \end{array}$$

If $m\in M_{2}^{\left(3\right)},$ then

$$\begin{array}{c} F_{j_{1},j_{2}:n}\left(x_{1},x_{2}\right)\leq 1 \end{array}$$

as the restriction of $\mathrm{uenv}{\tilde{M}}_{2}$ to $M_{2}^{\left(3\right)}$ equals ${\tilde{U}}_{2}^{\left(3\right)}$ and the equation of the plane passing through the points of ${\tilde{U}}_{2}^{\left(3\right)}$ has the form: $z_{3}=1.$

Next, we derive the lower bounds. It is evident that $M_{2}=M_{2}^{\left(4\right)}\cup M_{2}^{\left(5\right)}$ and the lower envelope $\mathrm{lenv}{\tilde{M}}_{2}$ of ${\tilde{M}}_{2}$ is given by

$$\begin{array}{c} \mathrm{lenv}{\tilde{M}}_{2}={\tilde{L}}_{2}^{\left(1\right)}\cup {\tilde{L}}_{2}^{\left(2\right)}, \end{array}$$

where

$$\begin{array}{ccc} M_{2}^{\left(4\right)} & = & \mathrm{conv}\{u^{\left(0\right)},u^{\left(2\right)},u^{\left(6\right)},u^{\left(7\right)}\},\\ M_{2}^{\left(5\right)} & = & \mathrm{conv}\{u^{\left(1\right)},u^{\left(6\right)},u^{\left(7\right)}\},\\ {\tilde{L}}_{2}^{\left(1\right)} & = & \mathrm{conv}\{\tilde{\Theta }\left(u^{\left(0\right)}\right),\tilde{\Theta }\left(u^{\left(2\right)}\right),\tilde{\Theta }\left(u^{\left(6\right)}\right),\tilde{\Theta }\left(u^{\left(7\right)}\right)\},\\ {\tilde{L}}_{2}^{\left(2\right)} & = & \mathrm{conv}\{\tilde{\Theta }\left(u^{\left(1\right)}\right),\tilde{\Theta }\left(u^{\left(6\right)}\right),\tilde{\Theta }\left(u^{\left(7\right)}\right)\}. \end{array}$$

The lower envelope of ${\tilde{M}}_{2}$ for n=10, $j_{1}=3$, and $j_{2}=5$ is illustrated in blue in Figure 1.

We consider two cases. If $m\left(t\right)\in M_{2}^{\left(4\right)},$ then ${\tilde{L}}_{2}^{\left(1\right)}$ is the restriction of $\mathrm{lenv}{\tilde{M}}_{2}$ to $M_{2}^{\left(4\right)},$ and points of ${\tilde{L}}_{2}^{\left(1\right)}$ satisfy the equation

$$z_{3}=0$$

Hence,

$$\begin{array}{c} F_{j_{1},j_{2}:n}\left(x_{1},x_{2}\right)\geq 0. \end{array}$$

If $m\left(t\right)\in M_{2}^{\left(5\right)},$ then

$$\begin{array}{c} F_{j_{1},j_{2}:n}\left(x_{1},x_{2}\right)\geq \frac{\left(n-j_{2}+1\right)nF\left(x_{1}\right)+\left(j_{2}-j_{1}\right)nF\left(x_{2}\right)-\left(j_{2}-1\right)\left(n-j_{1}+1\right)}{\left(n-j_{1}+1\right)\left(n-j_{2}+1\right)} \end{array}$$

because ${\tilde{L}}_{2}^{\left(2\right)},$ the restriction of $\mathrm{lenv}{\tilde{M}}_{2}$ to $M_{2}^{\left(5\right)},$ lies in the plane

$$\left(n-j_{1}+1\right)\left(z_{1}-n\right)+\left(j_{2}-j_{1}\right)z_{2}-\left(n-j_{1}+1\right)\left(n-j_{2}+1\right)\left(z_{3}-1\right)=0.$$

Summarizing, we have

$$\begin{array}{c} \max\left\{H_{j_{1},j_{2}:n}\left(F\left(x_{1}\right),F\left(x_{2}\right)\right),0\right\}\leq F_{j_{1},j_{2}:n}\left(x_{1},x_{2}\right)\leq \min\left\{\frac{n}{j_{1}}F\left(x_{1}\right),\frac{n}{j_{2}}F\left(x_{2}\right),1\right\} \end{array}$$

with $H_{j_{1},j_{2}:n}$ given by (14), and both the bounds are sharp. □

Now, we derive an analogue of Proposition 1 for the case of linear combinations of joint reliability functions of selected order statistics.

**Proposition 2.**  *Let F be a given distribution function. Let $1\leq j_{1}<j_{2}\leq n$ and $x_{1}<x_{2}$ be such that $0<F\left(x_{1}\right)<F\left(x_{2}\right)<1.$ If $X_{1},\ldots ,X_{n}$ are possibly dependent random variables with distribution function F, then*

$$\begin{array}{c} {\bar{F}}_{j_{1},j_{2}:n}\left(x_{1},x_{2}\right)\leq \min\left\{\frac{n\bar{F}\left(x_{1}\right)}{n-j_{1}+1},\frac{n\bar{F}\left(x_{2}\right)}{n-j_{2}+1},1\right\}, \end{array}$$

*and*

$$\begin{array}{c} {\bar{F}}_{j_{1},j_{2}:n}\left(x_{1},x_{2}\right)\geq \max\left\{{\bar{H}}_{j_{1},j_{2}:n}\left(\bar{F}\left(x_{1}\right),\bar{F}\left(x_{2}\right)\right),0\right\}, \end{array}$$

*where $\bar{F}=1-F$ and*

$$\begin{array}{c} {\bar{H}}_{j_{1},j_{2}:n}\left(z_{1},z_{2}\right)=1-\frac{n\left(j_{2}-j_{1}\right)\left(1-z_{1}\right)-nj_{1}\left(1-z_{2}\right)}{j_{1}j_{2}}. \end{array}$$

*The bounds are sharp.*

**Proof.**  Set k=1, q=2 and n≥2. Let F, $1\leq j_{1}<j_{2}\leq n,$ and $x_{1}<x_{2}$ be such that $0<F\left(x_{1}\right)<F\left(x_{2}\right)<1.$ Further, let ϑ, $I_{1}^{2}$, $\delta^{1}$, $\delta^{2},$ $U_{n}^{2}$, t, m(t), $\theta_{2}\left(u\right),$ and $M_{2}$ be as defined in the proof of Proposition 1. Clearly, $m\left(t\right)\in M_{2}.$

By Theorem 2, it suffices to find the values $C_{\bar{\varphi }}\left(m\left(t\right)\right)$ and $C^{\bar{\varphi }}\left(m\left(t\right)\right),$ where $\bar{\varphi }\left(u_{0},u_{1}\right)=1\left(u_{0}<j_{1},u_{0}+u_{1}<j_{2}\right).$ To this end, we determine the lower and upper envelopes of the set

$${\tilde{M}}_{2}=\mathrm{conv}\left\{{\tilde{\theta }}_{2}\left(u\right):u\in U_{n}^{2}\right\}$$

with ${\tilde{\theta }}_{2}\left(u_{0},u_{1}\right)=\left(u_{0},u_{1},\bar{\varphi }\left(u_{0},u_{1}\right)\right).$

It is easily seen that the set $\mathrm{ext}{\tilde{M}}_{2}$ of extreme points of ${\tilde{M}}_{2}$ equals

$$\{{\tilde{\theta }}_{2}\left(u^{\left(0\right)}\right),{\tilde{\theta }}_{2}\left(u^{\left(1\right)}\right),\ldots ,{\tilde{\theta }}_{2}\left(u^{\left(7\right)}\right)\},$$

in which

$$\begin{array}{c} u^{\left(0\right)}=\left(0,0\right),\;\;\;u^{\left(1\right)}=\left(n,0\right),\;\;u^{\left(2\right)}=\left(0,n\right),\;\;\;u^{\left(3\right)}=\left(j_{1}-1,0\right),\\ u^{\left(4\right)}=\left(j_{1}-1,j_{2}-j_{1}\right),\;\;\;u^{\left(5\right)}=\left(0,j_{2}-1\right),\;\;\;u^{\left(6\right)}=\left(j_{1},0\right),\;\;\;u^{\left(7\right)}=\left(0,j_{2}\right), \end{array}$$

that is,

$$\begin{array}{ccc} \mathrm{ext}{\tilde{M}}_{2} & = & \{\left(0,0,1\right),\left(n,0,0\right),\left(0,n,0\right),\left(j_{1}-1,0,1\right),\left(j_{1}-1,j_{2}-j_{1},1\right),\\ & & \;\;\left(0,j_{2}-1,1\right),\left(j_{1},0,0\right),\left(0,j_{2},0\right)\}. \end{array}$$

**Figure 2:**
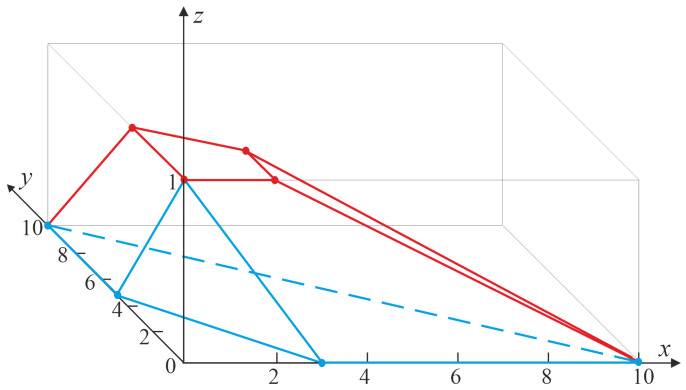
The set ${\tilde{M}}_{2}$ corresponding to ${\bar{F}}_{3,5:10}$.

Figure 2 shows the set ${\tilde{M}}_{2}$ corresponding to n=10, $j_{1}=3,$ and $j_{2}=5$.

We first derive the upper bounds. Observe that

$$M_{2}=M_{2}^{\left(1\right)}\cup M_{2}^{\left(2\right)}\cup M_{2}^{\left(3\right)}$$

and the upper envelope $\mathrm{uenv}{\tilde{M}}_{2}$ of ${\tilde{M}}_{2}$ is given by

$$\mathrm{uenv}{\tilde{M}}_{2}={\tilde{U}}_{2}^{\left(1\right)}\cup {\tilde{U}}_{2}^{\left(2\right)}\cup {\tilde{U}}_{2}^{\left(3\right)},$$

where

$$\begin{array}{ccc} M_{2}^{\left(1\right)} & = & \mathrm{conv}\left\{u^{\left(1\right)},u^{\left(3\right)},u^{\left(4\right)}\right\},\;M_{2}^{\left(2\right)}=\mathrm{conv}\left\{u^{\left(1\right)},u^{\left(2\right)},u^{\left(4\right)},u^{\left(5\right)}\right\},\\ M_{2}^{\left(3\right)} & = & \mathrm{conv}\{u^{\left(0\right)},u^{\left(3\right)},u^{\left(4\right)},u^{\left(5\right)}\} \end{array}$$

and

$$\begin{array}{ccc} {\tilde{U}}_{2}^{\left(1\right)} & = & \mathrm{conv}\{{\tilde{\theta }}_{2}\left(u^{\left(1\right)}\right),{\tilde{\theta }}_{2}\left(u^{\left(3\right)}\right),{\tilde{\theta }}_{2}\left(u^{\left(4\right)}\right)\},\\ {\tilde{U}}_{2}^{\left(2\right)} & = & \mathrm{conv}\{{\tilde{\theta }}_{2}\left(u^{\left(1\right)}\right),{\tilde{\theta }}_{2}\left(u^{\left(2\right)}\right),{\tilde{\theta }}_{2}\left(u^{\left(4\right)}\right),{\tilde{\theta }}_{2}\left(u^{\left(5\right)}\right)\},\\ {\tilde{U}}_{2}^{\left(3\right)} & = & \mathrm{conv}\{{\tilde{\theta }}_{2}\left(u^{\left(0\right)}\right),{\tilde{\theta }}_{2}\left(u^{\left(3\right)}\right),{\tilde{\theta }}_{2}\left(u^{\left(4\right)}\right),{\tilde{\theta }}_{2}\left(u^{\left(5\right)}\right)\}. \end{array}$$

The red outline in Figure 2 indicates the upper envelope of ${\tilde{M}}_{2}$ for n=10, $j_{1}=3$, and $j_{2}=5$.

We consider three cases. If $m\left(t\right)\in M_{2}^{\left(1\right)},$ then the third coordinate $C^{\bar{\varphi }}\left(m\left(t\right)\right)$ of the intersection point of the line {(m(t),ρ):ρ∈R} with the restriction ${\tilde{U}}_{2}^{\left(1\right)}$ of $\mathrm{uenv}{\tilde{M}}_{2}$ to $M_{2}^{\left(1\right)},$ which lies on the plane described by the equation

$$z_{1}+\left(n-j_{1}+1\right)z_{3}-n=0,$$

gives the desired bound:

$$\begin{array}{c} {\bar{F}}_{j_{1},j_{2}:n}\left(x_{1},x_{2}\right)\leq \frac{n(1-F\left(x_{1}\right))}{n-j_{1}+1}. \end{array}$$

If $m\left(t\right)\in M_{2}^{\left(2\right)},$ then the restriction of $\mathrm{uenv}{\tilde{M}}_{2}$ to $M_{2}^{\left(2\right)}$ equals ${\tilde{U}}_{2}^{\left(2\right)},$ and any triple $\left(z_{1},z_{2},z_{3}\right)\in {\tilde{U}}_{2}^{\left(2\right)}$ satisfies

$$z_{1}+z_{2}+\left(n-j_{2}+1\right)z_{3}-n=0,$$

Hence, the upper bound is

$$\begin{array}{c} {\bar{F}}_{j_{1},j_{2}:n}\left(x_{1},x_{2}\right)\leq \frac{n(1-F\left(x_{2}\right))}{n-j_{2}+1}. \end{array}$$

If $m\left(t\right)\in M_{2}^{\left(3\right)},$ then

$$\begin{array}{c} {\bar{F}}_{j_{1},j_{2}:n}\left(x_{1},x_{2}\right)\leq 1 \end{array}$$

because ${\tilde{U}}_{2}^{\left(3\right)}$ is the restriction of $\mathrm{uenv}{\tilde{M}}_{2}$ to $M_{2}^{\left(3\right)},$ and the plane passing through the points of ${\tilde{U}}_{2}^{\left(3\right)}$ satisfies $z_{3}=1.$

Next, we derive the lower bounds. Observe that

$$\begin{array}{c} M_{2}=M_{2}^{\left(4\right)}\cup M_{2}^{\left(5\right)} \end{array}$$

and the lower envelope $\mathrm{lenv}{\tilde{M}}_{2}$ of ${\tilde{M}}_{2}$ is given by

$$\begin{array}{c} \mathrm{lenv}{\tilde{M}}_{2}={\tilde{L}}_{2}^{\left(1\right)}\cup {\tilde{L}}_{2}^{\left(2\right)}, \end{array}$$

where

$$\begin{array}{ccc} M_{2}^{\left(4\right)} & = & \mathrm{conv}\left\{u^{\left(1\right)},u^{\left(2\right)},u^{\left(6\right)},u^{\left(7\right)}\right\},\;M_{2}^{\left(5\right)}=\mathrm{conv}\left\{u^{\left(0\right)},u^{\left(6\right)},u^{\left(7\right)}\right\},\\ {\tilde{L}}_{2}^{\left(1\right)} & = & \mathrm{conv}\{\tilde{\theta }\left(u^{\left(1\right)}\right),\tilde{\theta }\left(u^{\left(2\right)}\right),\tilde{\theta }\left(u^{\left(6\right)}\right),\tilde{\theta }\left(u^{\left(7\right)}\right)\},\\ {\tilde{L}}_{2}^{\left(2\right)} & = & \mathrm{conv}\{\tilde{\theta }\left(u^{\left(0\right)}\right),\tilde{\theta }\left(u^{\left(6\right)}\right),\tilde{\theta }\left(u^{\left(7\right)}\right)\}. \end{array}$$

The blue outline in Figure 2 represents the lower envelope of ${\tilde{M}}_{2}$ for n=10, $j_{1}=3$, and $j_{2}=5$.

We consider two cases. If $m\left(t\right)\in M_{2}^{\left(4\right)},$ given the points in ${\tilde{L}}_{2}^{\left(1\right)},$ the restriction of $\mathrm{lenv}{\tilde{M}}_{2}$ to $M_{2}^{\left(4\right)}$ satisfy

$$z_{3}=0,$$

we have

$$\begin{array}{c} {\bar{F}}_{j_{1},j_{2}:n}\left(x_{1},x_{2}\right)\geq 0. \end{array}$$

If $m\left(t\right)\in M_{2}^{\left(5\right)},$ then

$$\begin{array}{c} {\bar{F}}_{j_{1},j_{2}:n}\left(x_{1},x_{2}\right)\geq 1-\frac{n\left(j_{2}-j_{1}\right)F\left(x_{1}\right)-nj_{1}F\left(x_{2}\right)}{j_{1}j_{2}} \end{array}$$

because the restriction ${\tilde{L}}_{2}^{\left(2\right)}$ of $\mathrm{lenv}{\tilde{M}}_{2}$ to $M_{2}^{\left(5\right)}$ lies in the plane

$$j_{2}z_{1}+j_{1}z_{2}+j_{1}j_{2}\left(z_{3}-1\right)=0.$$

The proof is complete. □

**Remark 5.**  *Proposition 2 provides the best-possible lower and upper bounds on the reliability of a pair of $\left(n-j_{1}+1\right)$-out-of-n and $\left(n-j_{2}+1\right)$-out-of-n systems based on common components with possibly dependent lifetimes.*

## Data Availability

No new data were created or analyzed in this study.
